# Neonatal‐Onset Chronic Granulomatous Disease Presenting With Recurrent Culture‐Negative Meningitis: A Case Report and Diagnostic Considerations

**DOI:** 10.1155/crii/1817159

**Published:** 2026-01-07

**Authors:** Anwar Abu Hetta, Rayyan G. Shakarnah, Khalil R. Salah, Jasem Y. Hroub, Abdallah N. Khatib, Amjad H. Rabei

**Affiliations:** ^1^ Department of Pediatrics, Alia Governmental Hospital, Hebron, State of Palestine; ^2^ Faculty of Medicine, Palestine Polytechnic University, Hebron, State of Palestine, ppu.edu; ^3^ Department of Clinical Medical Sciences, Faculty of Medicine and Health Sciences, Palestine Polytechnic University, Hebron, State of Palestine, ppu.edu

**Keywords:** case report, chronic granulomatous disease, culture-negative meningitis, dihydrorhodamine (DHR) test, primary immunodeficiency

## Abstract

Chronic granulomatous disease (CGD) is a primary immunodeficiency caused by defective phagocyte oxidative burst and may present in early life with severe or recurrent infections. We report a female infant born at 38 weeks’ gestation (birth weight 2700 g) who developed fever and presumed sepsis at 5 days of life, followed by multiple recurrent hospitalizations for febrile illness with suspected meningitis, diarrhea, dehydration, and failure to thrive. Cerebrospinal fluid (CSF) evaluations across episodes demonstrated pleocytosis with elevated protein and normal‐to‐low glucose, while Gram stain and CSF cultures were repeatedly negative, consistent with recurrent culture‐negative meningitis. Laboratory assessment showed intermittent anemia, thrombocytosis, and episodes of significant neutropenia. Complement and immunoglobulin levels were within reference ranges, and flow cytometry demonstrated preserved T‐ and B‐lymphocyte compartments. A flow cytometric dihydrorhodamine (DHR) oxidative burst assay was markedly abnormal (phorbol 12‐myristate 13‐acetate stimulation index 13%; *Escherichia coli* stimulation index 22.3%), supporting the diagnosis of CGD. At ~4.5 months of age, a sterile catheter urine culture grew multidrug‐resistant *Klebsiella pneumoniae* at ≥100,000 CFU/mL with susceptibility limited to aminoglycosides; the patient was treated with amikacin 15 mg/kg/dose intravenously once daily for 10 days, with defervescence within 48 h, clinical recovery, and a repeat urine culture showing no growth. Genetic testing was not performed due to financial and social constraints, and longer‐term outcomes beyond early infancy were unavailable in the record extract. This case underscores that recurrent culture‐negative meningitis in early infancy should prompt evaluation for primary immunodeficiency and that early DHR testing can expedite CGD diagnosis and guide timely preventive management and specialist referral.

## 1. Introduction

Chronic granulomatous disease (CGD) is an inherited defect of the phagocyte NADPH oxidase complex that impairs the respiratory burst and predisposes affected children to severe, recurrent bacterial and fungal infections, alongside inflammatory complications [1].

Although CGD is typically diagnosed in early childhood, symptomatic presentation in the neonatal period or early infancy is uncommon and may be misattributed to routine neonatal sepsis or meningitis, which can delay recognition of an underlying inborn error of immunity and timely preventive management [2, 3].

Recurrent meningitis with cerebrospinal fluid (CSF) pleocytosis and repeatedly negative cultures (“culture‐negative meningitis”) remains diagnostically challenging and carries a broad differential diagnosis; in infants with recurrent or unexplained episodes, clinicians should consider immunodeficiency and pursue a structured immunologic workup [4, 5].

The flow cytometry‐based dihydrorhodamine (DHR) oxidative burst assay provides a rapid functional assessment of NADPH oxidase activity and is central to confirming suspected CGD [2]. We report an infant with very early‐onset disease characterized by recurrent culture‐negative meningitis and failure to thrive, in whom a markedly abnormal DHR assay supported the diagnosis of CGD, followed by a multidrug‐resistant urinary infection managed with directed therapy. This case is presented to emphasize clear diagnostic reasoning, transparent reporting of microbiology and antimicrobial courses, and practical considerations for evaluating unexplained neonatal and infant infections [2, 4].

## 2. Case Presentation

A 5‐month‐old female infant was born at 38 weeks’ gestation via normal vaginal delivery (birth weight 2700 g) and did not require neonatal intensive care. The early neonatal course was notable only for mild jaundice. Maternal history included recurrent urinary tract infections during the third trimester and documented Group B Streptococcus (GBS) colonization; the intrapartum antibiotic prophylaxis status was not recorded in the available extracts. There was no known family history of primary immunodeficiency. The parents were consanguineous (paternal parallel cousins). Genetic testing to define the causative CGD variant and inheritance pattern was not performed due to financial and social constraints.

The infant first presented at 5 days of life with fever and decreased oral intake and was admitted for presumed neonatal sepsis. Thereafter, she experienced multiple recurrent admissions (≥7 documented episodes by history) for recurrent febrile illness with suspected meningitis, diarrhea, dehydration, and poor weight gain. During a later documented episode (age ~ 45 days), she was febrile (38.2°C, axillary), hypoactive, and had a bulging anterior fontanel; no focal neurological deficits were recorded, and the remainder of the examination was noncontributory. An earlier admission at ~ 14 days of age documented fever associated with bloody umbilical discharge.

Key clinical events, investigations, microbiology, and antimicrobial regimens are summarized in Table 1.

**Table 1:** Clinical timeline, investigations, microbiology, and antimicrobial therapy.

Age/date	Presentation	Key investigations (units)	Microbiology	Antimicrobial therapy (dose/route/interval/duration) and rationale	Outcome/notes
Day 5 of life	Fever; decreased oral intake; presumed sepsis	Sepsis evaluation ordered (CBC, CRP, renal/liver tests, urinalysis/urine culture, CSF analysis/culture, blood culture, and chest radiograph)	Culture results not available in extracted records	Empiric antibiotics planned after sampling; specific agents/dose/duration not captured	—

Day 14 of life	Fever (38.2°C) with bloody umbilical discharge; presumed sepsis	CSF and urine studies obtained	CSF culture: no growth; urine culture: negative	Ampicillin 175 mg IV every 8 h + gentamicin 14 mg IV every 24 h (duration not documented)	—

Day 14 (hospital Day 2)	Meningitis suspected	CSF WBC 30 cells/µL; lymphocytes 100%; protein 92.7 mg/dL; glucose 44 mg/dL	CSF culture: no growth	Escalated to meningeal dosing due to CSF pleocytosis: ampicillin 235 mg IV three times daily + cefotaxime (Claforan) 235 mg IV three times daily (duration not documented)	Clinical improvement documented

Day ~45	Recurrent fever; suspected sepsis/meningitis	Bulging anterior fontanel; sepsis evaluation repeated	Culture results not available in extract	Ampicillin + gentamicin started (dose/interval/duration not captured in available extract)	—

10–11 Jun 2025 (~3 months)	Fever with neutropenia; evaluated for immunodeficiency	CBC: WBC 4.25 × 10^9^/L; ANC 0.36 × 10^9^/L; Hb 9.67 g/dL; PLT 387 × 10^9^/L; lymphocyte subsets performed	Not documented	Antibiotics initiated with sepsis evaluation (agents/duration not captured); ANC later improved to 0.664 × 10^9^/L (per report)	—

24 Jun 2025	CGD evaluation	DHR oxidative burst (flow cytometry): PMA 13%; *E. coli* 22.3%	—	CGD supported; prophylaxis initiated under immunology care (agents/doses not captured); HSCT evaluation planned	—

15 Jul 2025 (~4.5 months)	Urinary infection episode	Urine culture (catheter specimen) and susceptibility	*K. pneumoniae* ≥100,000 CFU/mL (CRE); S: amikacin, gentamicin; R: carbapenems, cephalosporins, fluoroquinolones, TMP‐SMX.	Amikacin 15 mg/kg/dose IV once daily for 10 days (directed therapy)	Defervescence within 48 h; clinical resolution; repeat urine culture: no growth

Across documented admissions, CSF analyses demonstrated pleocytosis with elevated protein and normal‐to‐low glucose while Gram stain and CSF cultures were repeatedly negative, consistent with a culture‐negative meningitis pattern. Available CSF values included WBC 30 cells/µL with 100% lymphocytes, protein 92.7, and glucose 44 mg/dL and WBC 50 cells/µL with 90% lymphocytes/10% neutrophils, RBC 1000 cells/µL, protein 79.2, and glucose 42 mg/dL, with a repeat sample showing RBC 3000 cells/µL, protein 83.2 , glucose 36 mg/dL, and negative Gram stain. Systemic inflammation was supported by elevated C‐reactive protein in at least one episode (CRP 30.6; laboratory reference 0–5). Hematologic abnormalities included intermittent anemia, thrombocytosis, and episodes of significant neutropenia (e.g., ANC 0.36 × 10^9^/L at ~ 3 months, later improving to 0.664 × 10^9^/L per report).

Complement and immunoglobulins were within the reporting laboratory reference ranges. Flow cytometry demonstrated preserved T‐ and B‐cell compartments; lymphocyte subset immunophenotyping results are summarized in Table 2.

**Table 2:** Lymphocyte subset immunophenotyping (flow cytometry).

Parameter	Result	Unit	Reference range (reported)
Total WBC	4250	cells/mm³	3500–23,560
Lymphocytes	3426 (80.6)	cells/mm³ (%)	—
CD3 + T cells	2603 (76.0)	cells/mm³ (%)	60.0–85.0%
CD4 + T cells	2055 (60.0)	cells/mm³ (%)	36.0–63.0%
CD8 + T cells	548 (16.0)	cells/mm³ (%)	15.0–41.0%
CD19/CD20 + B cells	548 (16.0)	cells/mm³ (%)	5.0–25.0%
CD56/CD16 + cells	260 (7.0)	cells/mm³ (%)	6.0–30.0%
CD3 + CD56 + CD16 + cells	0	(%)	—

The immunophenotyping report did not specify the NK gating strategy (e.g., CD3−CD16/56+), and repeat immunophenotyping or NK functional testing was not available; therefore, NK‐related findings should be interpreted cautiously and not assumed to represent persistent NK‐cell deficiency.

A flow cytometric DHR oxidative burst assay was markedly abnormal (PMA stimulation index 13%; *Escherichia coli* stimulation index 22.3%), indicating severely impaired neutrophil oxidative burst and supporting the diagnosis of CGD. Genetic confirmation (gene/variant and inheritance pattern) was not performed due to financial and social constraints.

Empiric intravenous antimicrobials were administered during multiple admissions for suspected neonatal sepsis/meningitis. At 14 days of age, treatment included ampicillin 175 mg IV every 8 h plus gentamicin 14 mg IV every 24 h. On hospital Day 2 of the same admission, therapy was escalated to meningeal dosing with ampicillin 235 mg IV three times daily plus cefotaxime 235 mg IV three times daily due to CSF pleocytosis consistent with meningitis. For other admissions, antibiotic initiation was documented, but course‐level details (start/stop dates and total duration) were not consistently captured in the available extracts.

At ~4.5 months of age (15 July 2025), an aseptically collected urine specimen obtained via transurethral catheterization grew *Klebsiella pneumoniae* at ≥100,000 CFU/mL, reported as CRE (carbapenemase‐nonproducer). The isolate was susceptible to amikacin and gentamicin and resistant to carbapenems (imipenem, meropenem, and ertapenem), third‐/fourth‐generation cephalosporins (ceftriaxone, ceftazidime, and cefepime), fluoroquinolones (ciprofloxacin and levofloxacin), and trimethoprim–sulfamethoxazole (TMP–SMX). The infant was treated with amikacin 15 mg/kg/dose IV once daily for 10 days, with defervescence within 48 h, clinical resolution, and a repeat urine culture showing no growth.

Following CGD confirmation, antibacterial and antifungal prophylaxis were initiated under pediatric immunology care, and referral for hematopoietic stem cell transplantation (HSCT) evaluation was arranged; prophylaxis agents/doses were not captured in the available extracts.

At the most recent recorded follow‐up (~5 months of age), the infant was under ongoing pediatric immunology care and had been referred for HSCT evaluation. However, the available documentation did not include longitudinal follow‐up data (e.g., interim infectious events, growth parameters, repeat immunologic testing, tolerance/adherence to prophylaxis, or whether HSCT was performed); therefore, outcomes beyond this timepoint cannot be reported from the current record extract.

## 3. Discussion

This case describes a term female infant with very early‐onset severe infections beginning at Day 5 of life, recurrent meningitis‐like episodes with persistent CSF inflammation despite repeatedly negative Gram stain and CSF cultures in the available documentation, intermittent cytopenias including severe neutropenia, and failure to thrive, in whom a markedly abnormal DHR oxidative burst assay supported a functional diagnosis of CGD [2]. The principal contribution is the diagnostic pathway: recurrent, culture‐negative meningeal inflammation in early infancy prompted immunodeficiency evaluation and led to timely functional confirmation of CGD [2, 6].

Recurrent “culture‐negative meningitis” in infants has a broad differential diagnosis that includes partially treated bacterial meningitis (especially when antibiotics precede lumbar puncture), viral etiologies, tuberculosis, fungal infection, and noninfectious inflammatory meningitis syndromes [6, 7]. When CSF pleocytosis and elevated protein persist across multiple admissions, negative cultures should not be interpreted as exclusion of serious disease; recurrence and severity should prompt broadened testing (where feasible) and assessment of host susceptibility, including evaluation for inborn errors of immunity [7, 8].

CGD is caused by an inherited defect in the phagocyte NADPH oxidase complex resulting in impaired oxidative burst and reduced intracellular killing, which can manifest as severe or recurrent infections early in life [1, 3]. In this patient, DHR flow cytometry provided decisive functional evidence (markedly reduced stimulation indices) supporting the diagnosis despite preserved immunoglobulin levels and broadly preserved T‐ and B‐cell compartments on immunophenotyping [2]. Genetic testing was not performed due to financial and social constraints; therefore, the causative gene and inheritance pattern cannot be assigned, genotype‐linked severity statements should be avoided, and the value of molecular confirmation for counseling and recurrence‐risk estimation should be acknowledged [9].

Empiric escalation to meningitis‐appropriate antimicrobial therapy is clinically coherent in infants presenting with fever, bulging fontanel, and CSF pleocytosis [6]. However, complete antibiotic course‐level documentation (weights to calculate mg/kg/dose, start/stop dates and times, and explicit reasons for each change) was not consistently available for early admissions; this limitation should be stated explicitly while all available regimen details are reported exactly as documented in the manuscript timeline.

The later urinary tract infection episode provides a clearly documented microbiologic and stewardship‐relevant event. A sterile catheter urine culture grew *K. pneumoniae* ≥100,000 CFU/mL, reported as CRE (carbapenemase nonproducer) with susceptibility limited to aminoglycosides, and the infant received amikacin 15 mg/kg/dose IV once daily for 10 days with defervescence within 48 h, clinical recovery, and repeat urine culture showing no growth [10, 11]. While a single case cannot establish comparative effectiveness, it illustrates the pragmatic role of susceptibility‐directed therapy for urinary‐source CRE infection when options are constrained [12].

NK‐cell‐related findings require cautious interpretation because CD56 is not exclusively NK‐specific and robust reporting depends on a stated gating strategy (commonly CD3−[CD16/56]+) and confirmation by repeat immunophenotyping and/or NK functional testing [13, 14]. In this case, the gating strategy was not specified in the available report extract, and repeat/functional NK testing was not documented; therefore, the NK‐related observation should be presented as non‐definitive and not assumed to represent persistent NK‐cell deficiency [15].

Strengths of this report include early functional confirmation of CGD with DHR flow cytometry, a recurrent CSF inflammatory pattern that prompted appropriate diagnostic escalation, and a well‐characterized CRE UTI with documented targeted treatment response and microbiologic clearance [2, 10]. Limitations include incomplete early microbiology (including the initial Day‐5 episode and maternal intrapartum prophylaxis status), incomplete antibiotic course details for some admissions, absence of genetic confirmation, incomplete NK‐cell methodological detail with no repeat/functional testing, and limited follow‐up beyond early infancy with HSCT evaluation pending [6, 9]. Overall, this case reinforces that recurrent culture‐negative meningitis‐like illness in early infancy—particularly with very early onset and systemic features—should prompt evaluation for primary immunodeficiency and that DHR testing can expedite diagnosis and guide timely preventive management and specialist referral [2, 16].

## Consent

Written informed consent for publication of this case report and any accompanying images was obtained from the patient’s parent/guardian. A copy of the consent form is held by the authors and is available for review by the journal on request.

## Disclosure

The study was performed as part of the employment/academic activity of the authors.

## Conflicts of Interest

The authors declare no conflicts of interest.

## Funding

This research received no specific grant from any funding agency in the public, commercial, or not‐for‐profit sectors.

## Data Availability

The data that support the findings of this study are available upon request from the corresponding author. The data are not publicly available due to privacy or ethical restrictions.
